# Meta-inflammation through the lens of macrophage programming and nutrient-sensing ghrelin signaling

**DOI:** 10.1097/IN9.0000000000000088

**Published:** 2026-08-06

**Authors:** Hye Won Han, Da Mi Kim, Sahar Eshghjoo, Chenxi Xu, Zeyu Liu, Bhimanagouda S. Patil, Shaodong Guo, Yuxiang Sun

**Affiliations:** 1 Department of Nutrition, Texas A&M University, College Station, TX, USA; 2 Agilent Technologies, Santa Clara, CA, USA; 3 Department of Horticultural Sciences, Texas A&M University, College Station, TX, USA

**Keywords:** ghrelin, growth hormone secretagogue receptor, macrophage reprogramming, polarization, infiltration, immunometabolism, meta-inflammation

## Abstract

Macrophages are at the critical interface of immunity and metabolism. Metabolic reprogramming of macrophages, also known as meta-inflammation, has major implications in the pathogenesis of many chronic diseases. The infiltration and polarization of macrophages play critical roles in tissue homeostasis and inflammatory responses, thereby reshaping the tissue microenvironment. Macrophages have unique phenotypical and functional plasticity, making them attractive as therapeutic targets. Nutrient-sensing ghrelin is a gastrointestinal peptide hormone that functions through its receptor, growth hormone secretagogue receptor (GHSR), and is known to trigger hunger sensation, stimulate food intake, and promote fat deposition. Emerging evidence indicates that ghrelin/GHSR signaling is also a critical regulator of immunometabolism, modulating metabolic pathways in macrophages to enable their adaptation and functional responses to the tissue microenvironment. Here, we highlight the regulatory mechanisms of the ghrelin/GHSR system in macrophage reprogramming in meta-inflammation. In particular, we demonstrate the infiltration and differentiation of tissue-resident macrophages, and their functional impacts on the development and progression of metabolic and inflammatory dysfunctions. The dynamic and multifaceted roles of ghrelin/GHSR signaling in macrophage reprogramming could be leveraged to develop novel immunotherapies for meta-inflammatory conditions and diseases.

## 1. Introduction

Chronic inflammation is increasingly recognized as a key contributor to the development and progression of metabolic diseases. The conventional view is that immunity and metabolism are distinct biological processes carried out by distinct cells. Recent advances have revealed extensive crosstalk between immunity and metabolism, particularly in metabolic diseases where immune and metabolic pathways are closely interconnected. Meta-inflammation refers to chronic, metabolically triggered inflammation driven by immune dysregulation that contributes to metabolic dysfunction and tissue damage in diseases such as obesity, diabetes, and metabolic dysfunction–associated steatotic liver disease (MASLD) ^[1–4]^. Unlike acute inflammation, meta-inflammation is characterized by chronic low-grade immune activation that persists over prolonged periods and progressively impairs tissue function ^[2]^. Sustained metabolic stress caused by nutrient excess, altered lipid metabolism, mitochondrial dysfunction, and gut-derived inflammatory signals can drive maladaptive immune responses that accelerate metabolic disease progression ^[2]^. Although multiple immune cells participate in tissue damage and repair ^[5]^, macrophages are particularly important because their remarkable functional versatility allows them to regulate inflammatory progression and tissue repair across diverse organ systems ^[6]^.

Macrophages are a crucial source of inflammatory mediators, including chemokines and cytokines, which elicit their effects in an autocrine and/or paracrine manner, driving the initial cellular response to tissue injury ^[6,7]^. Under metabolic stress, monocytes in the circulation infiltrate into the tissues to become macrophages, and macrophages undergo extensive metabolic reprogramming of glycolysis or oxidative phosphorylation (OXPHOS), thereby altering the types and levels of their cytokine production ^[8]^. These metabolic adaptations directly shape macrophage phenotype, with glycolysis promoting pro-inflammatory activation and oxidative metabolism supporting homeostasis and tissue repair ^[8]^. Therefore, understanding the molecular signals that regulate macrophage metabolic programming is critical for elucidating the mechanisms underlying meta-inflammation.

In addition to intracellular metabolic pathways, emerging evidence indicates that systemic metabolic hormones can directly regulate immune cell function. Hormones such as insulin, leptin, adiponectin, and glucagon-like peptide-1 (GLP-1) have been shown to modulate immune cell activation, inflammatory signaling, and cellular metabolism, highlighting the close interplay between endocrine regulation and immune regulation in metabolic diseases ^[9–11]^. Ghrelin, an endogenous ligand for the growth hormone secretagogue receptor (GHSR), is the only known circulating orexigenic hormone that stimulates appetite and promotes obesity and insulin resistance ^[12–14]^. GHSR is highly expressed in the brain but is expressed at much lower levels in peripheral tissues ^[15,16]^. We and others have shown that GHSR is expressed in immune cells, including macrophages and monocytes ^[16–18]^. However, compared with other metabolic hormones, the role of ghrelin/GHSR signaling in macrophage programming and meta-inflammation remains significantly understudied. This review highlights recent advances in ghrelin/GHSR signaling at the interface of immunity and metabolism, with particular focus on the regulation and functional roles of macrophage GHSR in meta-inflammation.

## 2. Fate of macrophages: from bone marrow progenitor cells to tissue macrophages

### 2.1 Hematopoietic origin and differentiation of macrophages

Macrophages are specialized myeloid cells that are generated from committed hematopoietic stem cells (HSCs) located in the bone marrow. HSCs are maintained in the bone marrow before entering the circulation. HSCs initially give rise to multipotent progenitors, which subsequently differentiate into common lymphoid and myeloid progenitors. These progenitors further differentiate into megakaryocyte–erythroid progenitors and granulocyte–macrophage progenitors ^[19–21]^. Granulocyte–macrophage progenitors then give rise to granulocytes as well as macrophage and dendritic cell precursors, which further differentiate into dendritic cell precursors and monoblasts (precursors of pro-monocytes) ^[20,21]^. Pro-monocytes then continue to differentiate into monocytes, which are subsequently released into the bloodstream ^[22]^.

After leaving the bone marrow, monocytes circulate in the bloodstream for several days. A subset of these cells subsequently extravasates across the endothelium and infiltrates peripheral tissues mediated by adhesion molecules such as L-selectin (CD62L), P-selectin glycoprotein ligand-1, lymphocyte function–associated antigen-1, and platelet and endothelial cell adhesion molecule-1, as well as chemotactic factors including colony-stimulating factor 1 (CSF1) and chemokines such as C-X3-C motif chemokine ligand 1 (CX3CL1) and CXCL12 ^[23–25]^. Once in the tissues, monocytes differentiate into infiltrating macrophages in response to CSF1 and chemoattractant-medicated inflammatory stimuli. Macrophage differentiation within tissues is tightly regulated by growth factors and transcription factors, including Notch1 and runt-related transcription factor 1 ^[26,27]^.

### 2.2 Tissue macrophage heterogeneity and metabolic reprogramming

Tissue macrophages comprise monocyte-derived infiltrating macrophages and tissue-resident macrophages, which together coordinate immune defense and maintain tissue homeostasis ^[6,28]^. While infiltrating macrophages are derived from circulating monocytes and are often found under pathological conditions, such as obesity and metabolic syndrome ^[29,30]^, tissue-resident macrophages are derived from progenitor cells from the yolk sac during development and maintain tissue homeostasis under steady state ^[31,32]^. Tissue-resident macrophages are present in various tissues and are referred to by distinct names, such as microglia in the brain, Kupffer cells in the liver, alveolar macrophages in the lung, osteoclasts in the bone, and splenic, adipose, and cardiac macrophages ^[33–35]^.

Recent studies have highlighted the diverse phenotypes and functions of tissue macrophages across organs ^[36]^. Tissue-resident macrophages are shaped by local tissue-derived signals and acquire specialized functions that reflect their developmental origin, enabling them to maintain tissue homeostasis ^[36]^. Microglia regulate neuronal development and synaptic pruning in the brain, Kupffer cells maintain immune surveillance and lipid metabolism in the liver, alveolar macrophages facilitate surfactant clearance in the lung, and adipose tissue macrophages contribute to lipid buffering and metabolic homeostasis ^[37–41]^. In obesity, excessive nutrient and lipid accumulation together with increased gut-derived endotoxin exposure promote the expansion of pro-inflammatory macrophages that contribute to inflammation and insulin resistance ^[40,41]^. Notably, aging is associated with persistent activation of tissue-resident macrophages, which drives meta-inflammation and further exacerbates chronic, low-grade inflammation, commonly referred to as inflammaging ^[40,41]^.

### 2.3 Tissue-resident macrophages in tissue homeostasis and immune activation state

Although tissue-resident macrophages do not migrate far from their local niches, they have critical roles in regulating local tissue remodeling, maintaining tissue homeostasis, and reshaping the microenvironment in disease states. Some studies examine macrophage activation in vitro by studying endotoxin-induced cytokine gene expression/secretion, and other studies focus on macrophage responsiveness in vivo and the intricate interactions between tissue-resident macrophages and other immune cells ^[31,42]^. These tissue-resident macrophages not only engulf foreign substances but also promote the recruitment of circulating monocytes to inflamed tissues, thereby enhancing the population of infiltrating macrophages ^[43]^. In the abdominal cavity, macrophages clear dead cells by dismantling them into smaller particles and recruit peritoneal macrophages and neutrophils to the injured site within an hour ^[44]^. In tissues such as the liver, heart, and intestine, tissue-resident macrophages become activated and release pro-inflammatory cytokines during the early phase of injury and inflammation, which in turn recruit more circulating monocytes to the injured tissues ^[45]^.

The effectiveness of recruiting circulating immune cells into the tissues is dependent on cytokines and chemokines ^[46]^. Bone marrow–derived monocytes have two subtypes: type 1 monocytes have high expression of C‑X3‑C motif chemokine receptor 1 (CX3CR1) and low expression of lymphocyte antigen 6 complex, locus C (LY6C), and do not respond to the C-C motif chemokine ligand 2 (CCL2); type 2 monocytes have low CX3CR1 expression and high LY6C expression, enabling them to respond to CCL2 ^[47,48]^. Under conditions of tissue injury, nucleotide-binding oligomerization domain-containing protein 2 (NOD2) in activated tissue-resident macrophages increases the production of CCL2, which promotes the recruitment of monocytes to the injured site and their differentiation into inflammatory macrophages ^[49]^. The CCL2-mediated pathway highlights the role of tissue-resident macrophages as the first line of defense against invading pathogens by coordinating communication between injured tissues and bone marrow–derived immune cell mobilization, thereby enhancing macrophage recruitment and activation.

### 2.4 Microenvironmental regulation of tissue-resident macrophage specialization

During embryonic development, tissue-resident macrophages are defined by their expression of cell surface markers, such as FcγRI and MERTK, and transcription factors, including purine-rich box 1 (PU.1), CCAAT/enhancer-binding proteins (C/EBPs), musculoaponeurotic fibrosarcoma (MAF), and musculoaponeurotic fibrosarcoma B (MAFB) ^[50,51]^. Interestingly, depending on their location, macrophages become specialized to respond to specific signals produced in the local microenvironment; this enables them to precisely regulate the expression of tissue-specific transcription factors according to the needs of the residing organ ^[52–54]^. In the brain, microglia are exposed to locally produced transforming growth factor-β (TGF-β), which activates the SMAD2/3 signaling pathway, leading to the expression of microglia-specific genes. For example, interleukin (IL)‑34, produced by neurons in various regions of the brain, governs microglial maintenance ^[55,56]^. Microglia interact with the surrounding environment and produce brain‑derived neurotrophic factor and insulin‑like growth factor 1, which are crucial for the survival of layer V cortical neurons ^[57]^. In the lung, fetal monocytes differentiate into alveolar macrophages after being exposed to epithelial cell-derived colony-stimulating factor 2, which promotes the expression of peroxisome proliferator‑activated receptor gamma (PPARγ) ^[58]^. Mature alveolar macrophages are essential for maintaining lung homeostasis by clearing excess surfactant and regulating its turnover. In the spleen, the expression of the transcription factor SPI-C is regulated by heme, which is required for the differentiation and maintenance of red pulp macrophages. These macrophages express the adhesion molecule vascular cell adhesion molecule 1 and play a critical role in iron recycling and the clearance of senescent erythrocytes ^[59]^. Splenic macrophage maintenance depends on liver X receptor alpha (LXRα)-mediated signals to trap circulating particulates to support local B-cell maintenance in the marginal zone of the spleen ^[60,61]^. Analogously, in the intestine, microbial products in the intestine drive the production of IL-1β, which stimulates group 3 innate lymphoid cells ^[62]^. Colony-stimulating factor 2 production mediated by group 3 innate lymphoid cells is required for the survival of intestinal immune cells (macrophages and dendritic cells), and their secreted IL-10 and retinoic acid are essential factors for intestinal T (Treg) cell homeostasis. In the liver, Kupffer cells are maintained by CSF1 signaling, which induces LXRα activation. LXRα, in turn, regulates genes involved in erythrocyte clearance and antigen removal from the portal circulation ^[63]^. In the skin, IL-34 and TGF-β produced by keratinocytes drive the expression of transcription factors in Langerhans cells, which promote skin tolerance and immunity ^[55,64]^. Lastly, cardiac macrophages are activated and maintained by locally secreted CSF1, which supports their survival and functional interactions with cardiomyocytes ^[65]^. However, the precise mechanisms by which CSF1 signaling regulates cardiomyocyte homeostasis remain unclear.

## 3. Macrophage reprogramming and functional adaptation in meta-inflammation

### 3.1 Recruitment and accumulation of macrophages during metabolic stress

Tissue macrophages are important for maintaining tissue homeostasis ^[66,67]^. While resident macrophages control homeostasis in the tissue under steady state ^[68,69]^, infiltrating macrophages are found under pathological conditions, such as obesity and metabolic syndrome ^[30,70]^. It has been reported that blockade of macrophage infiltration inhibits activation of tissue-resident macrophages, leading to suppression of tissue injury ^[71]^. In the liver, depletion of resident Kupffer cells has been shown to reduce neutrophil recruitment, which demonstrates that Kupffer cells play an important role in the healing of liver injury due to neutrophil mobilization ^[72]^. However, the roles of tissue macrophages in tissue injury are complex; both deleterious and protective functions have been reported ^[36,73,74]^. This discrepancy could be a result of the heterogeneity of tissue macrophages and the difficulty in identifying unique cell markers for resident and infiltrating macrophages. To that end, recent progress in multiparameter flow cytometry has enhanced the capability of distinguishing different macrophage subsets, which helps to unravel the complexity of the distinctive macrophages in different organs ^[75–77]^.

### 3.2 Macrophage polarization and metabolic reprogramming

Macrophages play a dynamic role in the maintenance of tissue homeostasis as well as in the activation of innate immunity under disease conditions ^[78]^. Macrophages have unique characteristics of being plastic and dynamic; they are highly responsive to stimuli and insults in the tissue microenvironment, thus are considered “gate keepers". Traditionally, macrophage polarization is defined as classically M1-like and M2-like subtypes according to their apparent phenotypes and functions. Classically activated macrophages, induced by lipopolysaccharide and/or interferon-gamma, exhibit increased glycolysis and reduced OXPHOS, produce pro-inflammatory cytokines, and play key roles in infectious and inflammatory diseases ^[79]^. By contrast, M2-like macrophages, activated by IL-4 and/or IL-13, exhibit enhanced OXPHOS, produce anti-inflammatory cytokines, and are often associated with wound healing and immunomodulation ^[80–82]^. Polarized macrophages reprogram their metabolic signaling pathways, utilizing glycolysis or mitochondrial OXPHOS ^[82–84]^. Traditionally, the activation of macrophages in chronic or acute inflammatory diseases has been described as M1 or M2 activation based on metabolic characteristics in the literature ^[80,85]^. While the M1–M2 paradigm has been informative for describing macrophages in vitro, it is now recognized as insufficient to accurately capture the functional diversity of macrophages in vivo. Tissue macrophages have much greater heterogeneity, presenting as a continuous spectrum of different subtypes, rather than simply pro-inflammatory M1 or anti-inflammatory M2 macrophages ^[86]^. In vivo, microenvironmental signals continuously shape macrophage activation, generating diverse intermediate and tissue-specific states rather than two mutually exclusive phenotypes ^[86]^.

### 3.3 Macrophage phagocytosis and efferocytosis in meta-inflammation

Macrophages maintain tissue homeostasis primarily through phagocytosis of pathogens and debris, and efferocytosis of apoptotic cells. During phagocytosis, macrophages recognize targets via Fc, complement, and pattern-recognition receptors, triggering actin remodeling and engulfment into phagosomes that mature through endosomal acidification and fusion with enzyme-rich lysosomes ^[87,88]^. Reactive oxygen and nitrogen species produced during this process further enhance the antimicrobial activity of macrophages ^[89–93]^. During efferocytosis, apoptotic cells expose phosphatidylserine as an “eat-me” signal, which engages specialized receptors and initiates noninflammatory internalization. Fusion of efferosomes with lysosomes completes the degradation of apoptotic cells, preventing secondary necrosis and sustaining tissue homeostasis ^[94–96]^.

Under meta-inflammatory states, such as obesity, insulin resistance, and diabetes, excess nutrient availability and lipotoxicity impair these clearance programs. Cholesterol and saturated fatty acids destabilize lipid rafts, impair receptor clustering, and decrease membrane flexibility ^[97–100]^. Hyperglycemia and chronic activation of mechanistic target of rapamycin complex 1 suppress autophagy by inhibiting unc-51-like autophagy-activating kinase 1 and transcription factor EB signaling, thereby reducing lysosomal biogenesis and degradative capacity ^[101]^. In parallel, endoplasmic reticulum stress and reactive oxygen species disrupt vesicle trafficking and phagolysosomal fusion ^[102–105]^, while insulin signaling defects in the phosphoinositide 3-kinase (PI3K)/protein kinase B (Akt) pathway impair actin remodeling needed for phagocytic cup formation ^[106–109]^.

These combined insults result in defective engulfment and incomplete degradation, causing the accumulation of apoptotic bodies, lipid droplets, and cellular debris. The retained release of danger-associated molecular patterns activates Toll-like and other pattern-recognition receptors, as well as nuclear factor kappa‑light‑chain enhancer of activated B cells and c-Jun N-terminal kinases signaling, thus impairing macrophage function ^[110–114]^. Failure to clear the apoptotic cells also diminishes the generation of anti-inflammatory cytokines such as IL-10 and TGF-β, affecting resolution and tissue repair ^[41,70,115]^. Defective clearance has been implicated in many metabolic diseases, contributing to hepatic inflammation in MASLD, foam cell formation in atherosclerosis, insulin resistance in obesity, and persistent inflammatory signaling in type 2 diabetes ^[116,117]^. Thus, the impaired macrophage clearance represents a key driver of chronic inflammation and metabolic dysfunction.

### 3.4 Triggering receptor expressed on myeloid cells 2 (TREM2)-mediated adaptive macrophage responses in metabolic disease

A critical modulator of macrophage phagocytic adaptation of macrophages is TREM2. TREM2 recognizes anionic phospholipids, lipoproteins, and apoptotic-cell ligands, activating the DAP12–Syk–PI3K/AKT–mTOR and p38/MAPK–PPARγ pathways, which increase lipid uptake, efferocytosis, and mitochondrial respiration ^[118–129]^. Upon the stimulation of phagocytic signaling, macrophages differentiate into lipid-associated macrophages (LAMs) that surround lipid-rich/dying cells and buffer toxic lipids to promote anti-inflammatory gene programming ^[121–125]^. Loss of TREM2 impairs phagocytic clearance, heightens adipose and hepatic inflammation, and aggravates insulin resistance and glucose intolerance ^[120,128–133]^. Conversely, TREM2 activation restores phagocytosis, supports mitochondrial function, and promotes M2-like polarization ^[126–130]^. Thus, targeting TREM2 or restoring phagocytic function holds therapeutic promise for treating obesity-related metabolic diseases.

## 4. The roles of ghrelin/GHSR signaling in meta-inflammation

### 4.1 Ghrelin in energy metabolism

Ghrelin, a 28-amino acid peptide mainly secreted by the stomach, is an important orexigenic hormone, with hallmark functions including stimulating appetite, increasing food intake, and enhancing fat deposition. Under normal homeostatic conditions, plasma ghrelin levels fluctuate based on energy intake ^[9]^. Under negative energy balance, during fasting or before a meal, circulating ghrelin levels increase. Ghrelin levels fall in response to a meal; paradoxically, they are low in diet-induced obesity (DIO) ^[134]^. Several mechanisms have been proposed to explain the paradoxical suppression of ghrelin in over-nutritional states such as obesity, including ghrelin resistance as a physiological adaptation ^[135]^ and elevated insulin and leptin levels, inversely correlated with ghrelin, to compensate for reduced ghrelin levels ^[136,137]^. Research with gastric mucosal cells from obese mice showed reduced ghrelin production in response to physiological signals such as norepinephrine or glucose, suggesting that obesity desensitizes ghrelin-secreting cells ^[138,139]^. DIO increases duodenal somatostatin cells, which directly alter ghrelin secretion. Increased fatty acids and lactate in DIO can also decrease serum ghrelin levels by directly engaging short- and long-chain fatty acid receptors, G-protein-coupled receptors (GPCRs: GPR43 and GPR120), respectively, which are located on the plasma membrane of gastric ghrelin cells ^[140]^. Other studies reported that amino acids increase during obesity, activating a calcium-sensing receptor to decrease ghrelin release ^[141,142]^. Moreover, circulating levels of ghrelin do not decrease after the consumption of a meal in obese humans ^[143]^. These changes in ghrelin release may be due to ghrelin cells from obese mice possessing resistance to noradrenaline-mediated stimulation of the sympathoadrenal system, which is dependent on β1-adrenergic receptors ^[144]^. Consistently, ghrelin injection in obese mice fails to induce neuropeptide Y/agouti‑related peptide responsiveness, suggesting reduced neuropeptide Y/agouti‑related peptide-expressing neurons contribute to ghrelin resistance and suppression of the neuroendocrine ghrelin axis ^[145,146]^. In addition, ghrelin resistance in the hypothalamus observed in obese mice has been suggested to result from impaired transport of ghrelin across the blood–brain barrier ^[147]^.

### 4.2 Ghrelin/GHSR in macrophages

Ghrelin is ubiquitously expressed ^[148]^, but GHSR expression is more restricted; high expression of GHSR is detected in the neurons in the brain, and low levels are detected in a few peripheral tissues ^[149–151]^. We have reported that neuronal GHSR signaling affects neuroinflammation and metabolism-related behavioral changes through the AMP‑activated protein kinase–autophagy pathway ^[152]^. Ghrelin and GHSR are highly expressed in macrophages, and their expression increases during macrophage differentiation ^[153]^. Our recent work has provided direct evidence of GHSR involvement in macrophage programming under meta-inflammation, demonstrating its role in modulating inflammatory gene expression and macrophage polarization ^[18]^. More specifically, GHSR signaling metabolically programs macrophages through the protein kinase A (PKA)–cAMP response element-binding protein (CREB)–insulin receptor substrate 2 (IRS2)–protein kinase B beta (AKT2) signaling pathway, promoting pro-inflammatory M1-like polarization and contributing to impaired tissue insulin sensitivity polarization ^[18]^. Conversely, loss of GHSR promotes macrophage polarization toward an anti-inflammatory phenotype, resulting in attenuated inflammatory responses and improved metabolic homeostasis ^[18]^. Supporting this mechanism, ghrelin signaling has been shown to regulate insulin-associated signaling pathways and inflammatory responses in macrophages under both in vitro and in vivo conditions ^[154,155]^.

GHSR is also reported to regulate cholesterol release in human macrophages by activating ERK1/2 and PI3K/Akt signaling pathways, leading to PPARγ activation and subsequent upregulation of LXRα and the cholesterol transporters ABCA1 and ABCG1, which facilitate cholesterol efflux ^[154]^. Elevated cholesterol causes low-grade inflammation in humans ^[156]^, and the links between lipid metabolism and immune responses have been well documented ^[154,157]^. These observations suggest that ghrelin signaling may have important roles in immunity and lipid-mediated inflammation.

### 4.3 Ghrelin/GHSR signaling in meta-inflammation

Ghrelin, a stomach-derived orexigenic hormone and its receptor, GHSR, are traditionally recognized for their roles in energy metabolism. However, growing evidence suggests that both ghrelin and GHSR also play important roles in immunity and inflammation. A recent review discusses the evidence that ghrelin signaling is a pleiotropic anti‑inflammatory hormone in multiple organs, including the skin, nervous, cardiovascular, and hepatic systems ^[158]^. Similarly, Kratofil et al demonstrated that metabolic hormone signaling directly regulates monocyte function during tissue repair and identified monocyte-derived ghrelin, which functionally opposes leptin, as an important regulator that reduces vascular overgrowth and promotes post-infection healing ^[159]^. However, other studies indicate that its effects on inflammation are highly context‑dependent, particularly in acute endotoxemia and related short‑term inflammatory models, where the outcome varies with ghrelin dose, administration regimen, and disease stage ^[154,160,161]^. A rat skin injury study showed that repeated ghrelin administration (50–200 nmol/kg for 7 days) promoted wound healing in a dose-dependent manner, suggesting protective effects during acute tissue repair ^[162]^. Similarly, a cecal ligation and puncture–induced sepsis model demonstrated that ghrelin treatment (40 nmol/kg) reduces pulmonary oxidative stress and attenuates acute lung injury, supporting a protective role during early sepsis-induced inflammation ^[163]^. In contrast, in another cecal ligation and puncture–induced sepsis model, ghrelin (0.5 μg/g body weight, twice daily) shows a beneficial effect during the early phase, but prolonged treatment increased bacteremia in lean mice, with no effect observed in obese mice ^[164]^. These differential findings suggest that ghrelin-mediated immune regulation varies substantially depending on the inflammatory state, treatment duration, and metabolic context. At the cellular level, studies investigating the effects of ghrelin on inflammation have also reported mixed findings. Several studies have demonstrated anti-inflammatory effects of ghrelin in human monocytes and T cells, as well as modulating neuroinflammation following traumatic brain injury ^[160,165]^. In contrast, another study showed that ghrelin increased IL-8 production in GHSR-transgenic colonic epithelial cells ^[161]^.

**Figure 1.**
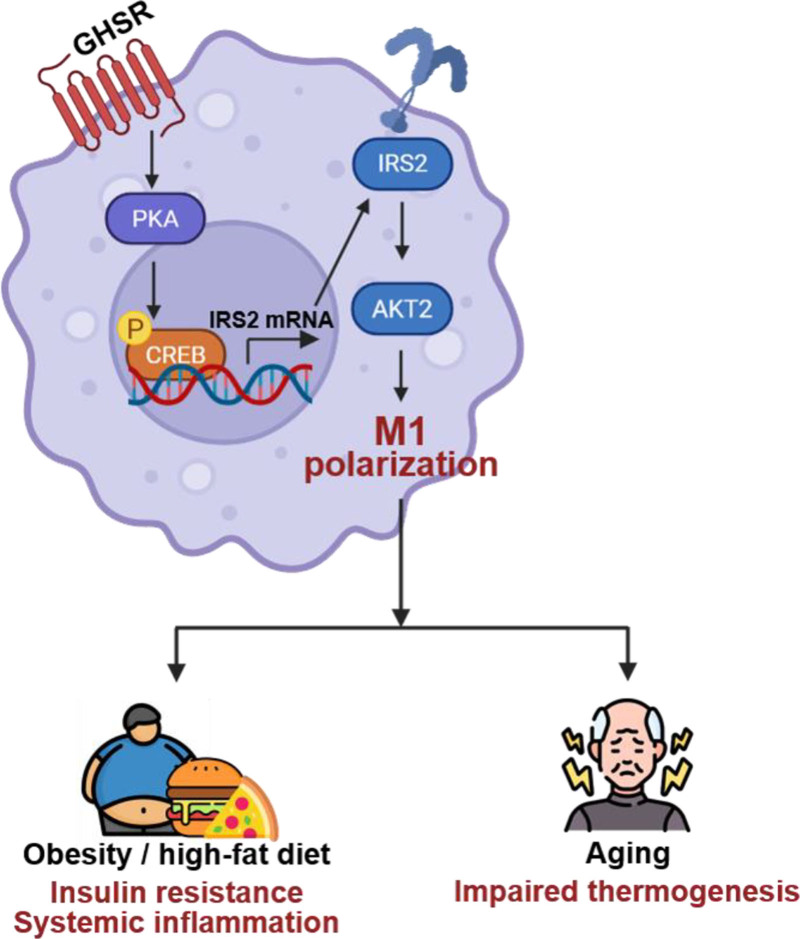
**Mechanisms of macrophage GHSR-mediated regulation of meta-inflammation and aging thermogenic impairment.** GHSR activation enhances PKA-dependent CREB phosphorylation, resulting in increased Irs2 transcription and downstream AKT2 activation. This signaling pathway promotes pro-inflammatory macrophage activation, thereby contributing to obesity-associated insulin resistance and aging-associated thermogenic impairment. GHSR, growth hormone secretagogue receptor. Created using BioLegend graphical resources.

Despite the well-established roles of GHSR signaling in metabolic regulation, its specific role in regulating meta-inflammation remains largely unexplored. In our recent study, myeloid-specific deletion of *Ghsr* attenuated systemic inflammation and improved insulin sensitivity under DIO, showing reduced infiltration of monocytes/macrophages, decreased pro-inflammatory macrophage activation, reduced lipid accumulation in adipose tissue and liver, and increased LAM populations ^[18]^. Mechanistically, GHSR promotes macrophage metabolic reprogramming toward a pro-inflammatory phenotype through the PKA–CREB–IRS2–AKT2 signaling pathway, suggesting that macrophage GHSR is a critical regulator of immunometabolic regulation ^[18]^. Furthermore, we investigated the role of macrophage GHSR in liver fibrosis and found that macrophage GHSR–FoxO1 signaling plays a key role in promoting liver fibrosis by enhancing inflammatory responses and activating hepatic stellate cells ^[166]^. Beyond direct evidence linking macrophage GHSR to meta-inflammation, additional studies suggest that GHSR signaling regulates broader immunometabolic pathways under metabolic stress. Adipose tissue-specific deletion of GHSR protected against high-fat DIO and insulin resistance by enhancing thermogenesis and modulating adipose tissue remodeling, supporting a role of GHSR in obesity-associated metabolic dysfunction ^[167]^. Similarly, our recent work showed that myeloid-specific deletion of *Ghsr* improved cold tolerance in aged mice, reduced pro-inflammatory macrophage remodeling in brown adipose tissue , and enhanced thermogenic activation ^[168]^. Together, these findings suggest that GHSR regulates immunometabolic adaptation under both obesity- and aging-associated inflammatory conditions (Figure 1).

The differential effects of ghrelin and GHSR in immunity and inflammation arise, in part, from the intrinsic properties of GHSR, a GPCR that has high constitutive (ligand-independent) activity ^[169]^ and regulates multiple downstream signaling pathways ^[170,171]^. GHSR can undergo heterodimerization with other GPCRs, further diversifying its signaling outputs. Consequently, the phenotypes observed following ghrelin administration may differ from those resulting from genetic or pharmacological manipulation of GHSR. For example, while ghrelin is known to induce the release of growth hormone and stimulate food intake via GHSR ^[172]^, ghrelin stimulates osteoblast growth and liver glucose production by mechanisms independent of GHSR ^[173–175]^. Therefore, it has been suggested that there may be unknown receptor(s) that bind ghrelin. GHSR, as a GPCR, has high constitutive activity that can be activated in the absence of ghrelin ^[169]^, which likely contributes to the discrepancies between ghrelin and GHSR. Indeed, we have reported that ghrelin null mice and GHSR null mice have differential thermogenic phenotypes and susceptibility to diet-induced inflammation ^[173,176,177]^. These findings highlight the complexity of the ghrelin/GHSR signaling axis in the regulation of meta-inflammation. More in-depth investigation is needed to further determine whether the effect of GHSR is pro- or anti-inflammatory under physiological and pathological conditions, as well as to decipher the effects at early and late stages of inflammation. Although the exact mechanisms are still being studied, growing evidence suggests that macrophage GHSR signaling plays a key role in macrophage programming during meta-inflammation and has the potential to serve as a therapeutic target for chronic metabolic and age-related inflammatory diseases.

## 5. Conclusions

Macrophages have important functions in meta-inflammation, which involve infiltration, polarization, and phagocytosis. Although infiltrating and tissue-resident macrophages arise from distinct origins, they function in concert to preserve tissue homeostasis and coordinately respond to injury or insult. Macrophages have unique phenotypic and functional plasticity, which makes them attractive therapeutic targets.

Emerging evidence supports that the nutrient-sensing ghrelin/GHSR signaling pathway plays important roles not only in metabolism but also in immunity. GHSR is a novel immune regulator of macrophages, with macrophage GHSR serving as a major driver of meta-inflammation. While ghrelin/GHSR represents a promising target for immunometabolism and has significant translational potential for immunometabolic diseases, the underlying molecular mechanisms are currently largely unknown. Future comprehensive studies are needed to further elucidate how ghrelin/GHSR regulates immune programming, thereby advancing the development of immunotherapies for meta-inflammatory diseases.

## Author contributions

Conceptualization: H.W.H., D.M.K., S.E., and C.X.; Literature review and synthesis: D.M.K., S.E., and C.X.; Writing—original draft preparation: D.M.K., S.E., and C.X.; Writing—Review & editing: H.W.H., Z.L., and B.S.P.; Supervision: Y.S.; Project administration: Y.S. H.W.H., D.M.K., S.E., and C.X. contributed equally to this paper. All authors have read and approved the final version of the manuscript.

## Conflicts of interest

The authors declare no conflicts of interest.

## Funding

This research was funded by CPRIT grants (RP250537 and RP250641), Texas A&M *Dementia & Alzheimer’s Research Initiative* (DARI), and in part by a grant from NIEHS (P30ES029067), Texas A&M AgriLife Institute for Advancing Health Through Agriculture (IHA) through the USDA Agreement No. 58-3091-1-018. Any opinions, findings, conclusions, or recommendations expressed in this publication are those of the authors and do not necessarily reflect the view of the NIH or USDA.
